# Study of the Influence of NanOx Parameters

**DOI:** 10.3390/cancers10040087

**Published:** 2018-03-21

**Authors:** Caterina Monini, Micaela Cunha, Etienne Testa, Michaёl Beuve

**Affiliations:** Institute of Nuclear Physics of Lyon (IPNL), Lyon 1 University, CNRS/IN2P3, 69622 Villeurbanne, France; cunha@ipnl.in2p3.fr (M.C.); testa@ipnl.in2p3.fr (E.T.); beuve@ipnl.in2p3.fr (M.B.)

**Keywords:** hadron therapy, biophysical modeling, NanOx, sensitivity study

## Abstract

NanOx is a new biophysical model that aims at predicting the biological effect of ions in the context of hadron therapy. It integrates the fully-stochastic nature of ionizing radiation both at micrometric and nanometric scales and also takes into account the production and diffusion of reactive chemical species. In order to further characterize the new framework, we discuss the meaning and relevance of most of the NanOx parameters by evaluating their influence on the linear-quadratic coefficient α and on the dose deposited to achieve 10% or 1% of cell survival, $D_{10\%}$ or $D_{1\%}$, as a function of LET. We perform a theoretical study in which variations in the input parameters are propagated into the model predictions for HSG, V79 and CHO-K1 cells irradiated by monoenergetic protons and carbon ions. We conclude that, in the current version of NanOx, the modeling of a specific cell line relies on five parameters, which have to be adjusted to several experimental measurements: the average cellular nuclear radius, the linear-quadratic coefficients describing photon irradiations and the α values associated with two carbon ions of intermediate and high-LET values. This may have interesting implications toward a clinical application of the new biophysical model.

## 1. Introduction

Hadron therapy is becoming an attractive modality for cancer treatment, as the exponential increase in the number of dedicated facilities built over the past decades shows. The favorable depth-dose profile of protons is mostly exploited to eradicate localized tumors situated close to organs at risk, while the high biological effectiveness makes carbon ion beams more adequate than the conventional radiotherapy modalities to treat radioresistant cancers. Such an efficiency in cell killing is quantified through the RBE (relative biological effectiveness), which is a complex function of multiple parameters related to the incident particles, the irradiation conditions and the intrinsic properties of the biological system. Determining the value of RBE for every scenario is a challenging task that requires modeling to comply with the demands of a clinical environment. Several solutions have already been developed (also specifically for protons, e.g., [1,2,3]), and a few are currently used in treatment planning [4,5,6,7]. However, the latter present some shortcomings that may limit their improvement: in the modified microdosimetric kinetic model, the nanometric scale is disregarded, and the Poissonian distribution relating cell survival to the total number of lethal damages is corrected in a second instance, as it is not adapted for high-LET ions; in the local effect model, the stochastic nature of the dose deposition is not taken into account at the nanoscopic level, and the use of the amorphous track structure results in conceptual incongruities [8,9]. In an attempt to overcome such drawbacks, we introduced a new biophysical model, NanOx, which allows one to calculate cell survival probability, taking into account the fluctuations in energy deposition at multiple scales and the production and diffusion of reactive chemical species. The description of the formalism, the results obtained for three cell lines, as well as the comparison with alternative models have previously been detailed in [10,11,12]. NanOx is based on a solid mathematical architecture, comprised of fundamental postulates, simplifications and approximations, which promotes the evolution and improvement of the model; the currently implemented version is the first one, as in [10,11,12].

This paper aims at analyzing the sensitivity of NanOx outcomes to its parameters. The relevance and influence of the latter are deduced quantitatively for three cell lines irradiated by monoenergetic protons and carbon ions: human tumor cells from salivary glands (HSG), lung fibroblasts (V79) and ovary (CHO-K1) cells from a Chinese hamster. In the following, we single out one parameter at a time, and we apply to it an input variation reproducing its experimental variability, or on the basis of theoretical arguments; keeping the standard values of all the remaining parameters, we evaluate three different outcomes. This procedure allows determining which are the parameters that need to be adjusted to experimental data and which are the ones that, showing a negligible dependence on the cell line, can be fixed according to specific considerations without altering the result in a significant way. As a consequence, this work provides a discussion of the input measurements required by the model and allows one to further characterize its simplified representation of the biophysical reality.

The basic principles of NanOx are pointed out in Section 2, together with an approximation introduced to accelerate computer calculations. Section 3 motivates the choice of the standard and modified values of the model parameters and describes the outcomes considered for the sensitivity analysis. The discussion and interpretation of the results, as well as the future perspectives resulting from this study, are approached in Section 4. Finally, some conclusions are drawn in Section 5.

## 2. Theoretical Framework

In order to integrate the stochasticity that characterizes ionizing radiation, NanOx includes dose fluctuations both at nanometric and micrometric scales, computing cell survival probability in terms of the average over all the configurations of radiation impacts and irradiated cells. A radiation impact corresponds to a primary particle interaction. It may consist of an ion track or of a photon interaction (e.g., photoelectric effect or Compton scattering), including all the secondary electrons and chemical species production. Ion track details and photon interactions are taken into account via the spatial distribution of the energy-transfer points and of the physico-chemical events created by *K* radiation impacts; such a configuration will be represented by index $c_{K}$ afterwards. At the present state, the communication between cells is considered implicitly included in cell response. Moreover, any effect due to the distribution in cell cycle or cell geometry is disregarded, and instead, a representative cell in an average cycle stage and with an average geometry is considered. As a result of the latter simplifications, the mean cell survival probability reads:

(1) $$\bar{S(D)}=\sum_{K=0}^{\infty }P\left(K,D\right)\;\cdot \;{\langle {}^{c_{K}}S_{}\rangle }_{c_{K}}$$

where P(K,D) is the probability to achieve *K* impacts with a delivered physical dose *D* and ${\langle {}^{c_{K}}S_{}\rangle }_{c_{K}}$ is the average survival probability over all the configurations $c_{K}$. NanOx attributes the process of cell death induction to the separate contributions of two types of biological events taking place at different spatial scales. The probability of cell survival, hence, may be decomposed into a factor due to the action of local lethal events ($S_{L}$) and one due to that of non-local events ($S_{\mathrm{NL}}$):

(2) $${}^{c_{K}}S_{}={}^{c_{K}}S_{L}\times {}^{c_{K}}S_{\mathrm{NL}}$$

The two terms appearing in Equation (2) are assumed to be independent and associated with two sensitive volumes that are, a priori, different since they are related to different biological events.

### 2.1. Local Lethal Events

Local lethal events are lethal events induced by physico-chemical processes in a very localized volume (<100 nm), inside which the probability that two or more particle tracks deposit a significant specific energy may be neglected at clinical doses [13]. They may correspond to the formation of complex DNA lesions (e.g., unrepaired/misrepaired DNA double-strand breaks) that may, on their own, lead to cell death. Local lethal events are modeled by the inactivation of one among *N* nanometric targets located in the sensitive volume. Such inactivation is described as a function of an observable characterizing the radiation quality at the local scale; for the current implementation of NanOx, we opted for the restricted specific energy, whose distribution is estimated from the LQD (LiQuiD water) Monte Carlo code [14]. This quantity is computed considering the energy transfers that may lead to events relevant for the biological effects of radiation (e.g., ionizations, excitations and attachments of electrons) and disregarding the energy that simply causes the heating of the medium (e.g., molecular vibrations, interactions between electrons and water phonons, geminate recombinations). We postulate that the responses of local targets are independent and that the probability of cell survival with respect to local lethal events for a given configuration of local targets ($c_{N}$) and radiation impacts ($c_{K}$) is equal to the probability that no local target is inactivated:

(3) $${}^{c_{N},c_{K}}S_{L}=\prod_{k=1}^{K}{}^{c_{N},c_{k}}S_{L}=\prod_{k=1}^{K}\prod_{i=1}^{N}\left(1-f({}^{c_{i},c_{k}}z_{})\right)$$

*f* represents the inactivation function, while ${}^{c_{i},c_{k}}z_{}$ is the restricted specific energy deposited in the local target *i* with configuration $c_{i}$ (i.e., position and orientation) after one radiation impact with configuration $c_{k}$. The introduction of an effective lethal function (ELF), defined by:

(4) F(x)=−Nln(1−f(x))

allows one to reformulate Equation (3) into:

(5) $${}^{c_{K}}S_{L}=\prod_{k=1}^{K}\exp\left(-{\langle F\left({}^{c_{i},c_{k}}z_{}\right)\rangle }_{c_{i}}\right)$$

provided that the number of targets (N) is large and that they are uniformly distributed. ${\langle F\left({}^{c_{i},c_{k}}z_{}\right)\rangle }_{c_{i}}$ represents the average effective lethal function over all the configurations of local targets.

### 2.2. Non-Local Events

Non-local events are harmful, but cannot induce cell death on their own. They represent the accumulation and the interaction, at the microscopic cellular scale, of sublethal damages (e.g., DNA single-strand breaks), lesions in different cellular structures (e.g., mitochondria, nuclear and cellular membranes) and oxidative stress. In the current version of NanOx, we decided to associate non-local events with *global events*, which account for the production of chemical reactive species in the associated sensitive volume. Indeed, it has been proven that the latter induce a significant part of DNA sublethal damages [15,16] and are directly involved in cellular oxidative stress. In order to properly characterize the radiation at the global scale, we introduced two new observables, which are evaluated via the LQD, PHYCHEML and CHEM Monte Carlo codes [14,17,18]. The relative chemical effectiveness (RCE) is determined as the ratio of the restricted specific energies deposited in the sensitive volume (*Z*) by the reference radiation and by a given ion causing the same level of oxidative stress; for the practical implementation of the model, we considered as the reference radiation low-LET photons emitted from a ^60^Co source, and we constructed RCE in terms of the chemical yields of ^•^OH radicals induced by such a reference radiation ($G_{r}$) and by the ion (*G*). Among all the radical species, ^•^OH molecules are characterized by the highest production rate and are considered the key reactants in DNA damage [19]. The chemical specific energy ($\tilde{Z}$), finally, corresponds to the specific energy rescaled by the factor RCE; for a configuration of radiation impacts $c_{K}$, it is defined as:

(6) $${}^{c_{K}}{\tilde{Z}}_{}=\sum_{k=1}^{K}{}^{c_{k}}{\mathrm{RCE}}_{}\;\cdot \;{}^{c_{k}}Z_{}=\sum_{k=1}^{K}\frac{{}^{c_{k}}G_{}}{G_{r}}\;\cdot \;{}^{c_{k}}Z_{}$$

Cell survival probability with respect to global events may be modeled via the well-known linear-quadratic (LQ) expression, but in terms of the chemical specific energy:

(7) $${}^{c_{K}}S_{G}\left({}^{c_{K}}{\tilde{Z}}_{}\right)=C_{\mathrm{norm}}\;\cdot \;\exp\left(-\alpha_{G}\;\cdot \;{}^{c_{K}}{\tilde{Z}}_{}-\beta_{G}\;\cdot \;{}^{c_{K}}{\tilde{Z}}_{}^{2}\right)$$

In Equation (7), $C_{\mathrm{norm}}$ is a normalization factor ensuring that the average cell survival probability over all irradiation configurations leads to the experimental cell survival with respect to a reference radiation characterized by the LQ coefficients $\alpha_{r}$ and $\beta_{r}$. The “global parameters” appearing in the same equation have been defined as follows, in the current version of the model: $\alpha_{G}$ is set as zero to allow for a separate modeling of the survival with respect to local lethal events; $\beta_{G}$ is instead derived from the $\beta_{r}$ parameter. It may be shown that:

(8) $$\beta_{G}=\frac{\beta_{r}}{\eta^{2}}$$

where coefficient η is the mean ratio between the restricted specific energy and the specific energy, estimated around 80% using the Monte Carlo code LQD.

### 2.3. Core and Penumbra Approximation

In order to simplify the implementation of the model and accelerate computer calculations, we exploited a feature that characterizes ion tracks: the presence of an inner core and an outer penumbra in which the energy deposition patterns are totally distinct. We designed the core as a parallelepiped with a 100-nm side centered along the ion track; this choice was motivated by the fact that, at therapeutic doses, the electrons reaching the penumbra may be represented, in a good approximation, in terms of the electrons excited and ionized by low-LET photons.

Such considerations allowed confining the tossing of nanometric targets to the volume $V_{c}$ describing the intersection between the track core and the sensitive volume, hence reducing the computing time required for the simulation of local lethal events. It is out of the scope of this paper to prove that Equation (5) may be reformulated as follows:

(9) $$\begin{array}{c} {}^{c_{K}}S_{L}\left({}^{c_{K}}Z_{}\right)=\prod_{k=1}^{K}\exp\left[-{}^{t_{k}}\alpha_{c}\frac{V_{c}}{V_{s}}\;\cdot \;{}^{t_{k}}Z_{c}-{}^{c_{k}}\alpha_{p}\frac{V_{p}}{V_{s}}\;\cdot \;{}^{c_{k}}Z_{p}\right] \end{array}$$

${}^{c_{k}}Z_{p}$ is the restricted specific energy calculated in the volume $V_{p}$ associated with the penumbra region after one radiation impact with configuration $c_{k}$; the corresponding coefficient ${}^{c_{k}}\alpha_{p}$ is set as the linear parameter describing the survival with respect to a reference radiation, $\alpha_{r}$. On the other hand, ${}^{t_{k}}Z_{c}$ is the average restricted specific energy calculated in $V_{c}$ over a large number of particles of type $t_{k}$; the coefficient ${}^{c_{k}}\alpha_{c}$ is determined for each radiation quality via the effective lethal function. It is worth noting that the various indices appearing in Equation (9) reflect the asymmetry in the approach used to model local lethal events arising from the core and the penumbra regions.

The core and penumbra approximation also allows one to express the cell survival probability with respect to global events in terms of the restricted specific energies and the chemical yields computed in the two complementary volumes (${}^{c_{k}}Z_{c/p}$ and ${}^{t_{k}}G_{c/p}$):

(10) $${}^{c_{K}}S_{G}\left({}^{c_{K}}Z_{}\right)=\prod_{k=1}^{K}C_{\mathrm{norm}}\;\cdot \;\exp\left[-\frac{\beta_{r}}{\eta^{2}}{\left(\frac{{}^{t_{k}}G_{c}}{G_{r}}\frac{V_{c}}{V_{s}}\;\cdot \;{}^{c_{k}}Z_{c}+\frac{{}^{t_{k}}G_{p}}{G_{r}}\frac{V_{p}}{V_{s}}\;\cdot \;{}^{c_{k}}Z_{p}\right)}^{2}\right]$$

Equation (10) is of particular interest when compared to Equation (7), since it clarifies the new concepts introduced to designate the chemical specific energy and summarizes the choices related to the current version of the model, as well. Further details on the formalism and on the choice of considering average observables for a given radiation type $t_{k}$ will be presented elsewhere.

### 2.4. Model Parameters

Several parameters have been introduced to estimate the cell survival probability in the description of the NanOx formalism. The modeling of local and global events is based on the definition of two critical cellular regions; in the current version of the model, both are assumed to correspond to the nucleus and are represented by the cylindrical volume $V_{s}$. The modeling of local lethal events relies on the effective lethal function *F*, which is represented by an error function built via a data-driven procedure:

(11) $$F\left(z\right)=\frac{h}{2}\left[1+\mathrm{erf}\left(\frac{z-z_{0}}{\sigma }\right)\right]$$

The three free parameters are determined through a fit to experimental α(LET) data, which are specific to a given cell line. In particular, $z_{0}$ represents the restricted specific energy threshold above which DNA damage may induce cell death, and σ is the width of the increase; *h*, the height of the response attesting to the function’s saturation, includes the total number of local targets (see Equation (4)). The simulation of *z* distributions is by definition based on the definition of convenient biological targets, which are defined as the cylindrical volumes $V_{t}$. The modeling of global events also requires the introduction of additional parameters, which appear explicitly or implicitly in Equation (7): $\alpha_{G}$, $\beta_{G}$ and the time $T_{\mathrm{RCE}}$ elapsed after the radiation impact. ^•^OH radicals diffuse, interact and recombine continuously with each other, so their concentration is a function of time.

## 3. Materials and Methods

We carried out a theoretical study on the influence of most of the parameters listed in Section 2.4 in the prediction of radiation-induced effects. In the current implementation of NanOx, we regarded as fixed both the size of local targets and the linear coefficient appearing in the description of cell survival probability with respect to global events, $\alpha_{G}$. The variation of such parameters will be the subject of future work.

### 3.1. Cell Lines and Standard Values of the Parameters

We considered three cell lines for which NanOx predictions have been already benchmarked against experimental data for photon, proton and carbon ion irradiations over a wide energy range, going from 0.8–266.4 MeV u^−1^ [10,12]. Human tumor cells from salivary glands (HSG) were chosen since head and neck cancers match the therapeutic indications for hadron therapy treatments, while normal Chinese hamster lung fibroblast (V79) and ovary (CHO-K1) cells were selected due to the large amount of data available in the literature. The analyzed cells were fairly radioresistant, the surviving fractions obtained after 2 Gy X-ray exposure being 0.42 for HSG, 0.65 for V79 and 0.58 for CHO-K1.

Several experimental input data were used to set up the standard configuration of the parameters required to model each cell line. First, the sensitive volume was defined as a cylinder with a radius ($R_{V_{s}}$) corresponding to the average nuclear size and a length ($L_{V_{s}}$) that was set to 1 μm. We considered that the latter represents the lowest reasonable value that can mimic the thickness of the nuclei of flattened cells and chose it for simplicity due to the scarcity of experimental measurements. Second, measured α values corresponding to radiation qualities of different types and LET allowed constructing the effective lethal function. The best combination of the $z_{0}$, σ and *h* parameters was determined via the Migrad minimization algorithm [20], using $z_{0}$ = 10,000 Gy, σ = 5000 Gy and *h* = 100,000 as initial conditions. Finally, as highlighted by Equation (8), $\beta_{G}$ was determined via the experimental $\beta_{r}$ coefficient. For each cell line, the choice of the reference radiation was based on the fact that both the linear and the quadratic components of a given photon irradiation were the closest to the average values over all the available publications. In the case of CHO-K1 cells, however, it was not possible to identify a measurement that corresponded to the “mean” ($\alpha_{r},\beta_{r}$) pair, so we applied a correction factor to the $\beta_{r}$ coefficient to reproduce the average value. The remaining parameters were fixed for all the cell lines according to specific considerations: the local targets were defined as cylinders with radius and length equal to 10 nm to mimic approximately the extension of a DNA DSB [13,21,22] and also take the diffusion of reactive species into account [21,23]; $\alpha_{G}$ was set as 0 to allow for a separate adjustment of local and global events; $T_{\mathrm{RCE}}$ was set as $10^{-11}$ s to represent the production of primary chemical reactive species [24]. Table 1 summarizes the standard values of the NanOx parameters chosen to characterize HSG, V79 and CHO-K1 cells.

### 3.2. Outcomes

NanOx’s main outputs are survival fractions, but to simplify the analysis, it is convenient to consider three complementary quantities as a function of the ions’ LET:

- the LQ parameter α, which is extensively used both experimentally and theoretically to assess the effect of different radiation qualities;
- $D_{10\%}$, the dose deposited to achieve 10% of cell survival, which is often used in clinical contexts;
- $D_{1\%}$, the dose deposited to achieve 1% of cell survival, which is necessarily more sensitive to the shoulder effect than the previous endpoint.

The LET values selected for the simulations correspond to the measurements available in the literature for each cell line; nonetheless, we will not show any experimental data in the sequel, since the goal of the present study is to clarify the relation between NanOx parameters and predictions.

### 3.3. Sensitive Volume, $V_{s}$

In order to assess the impact of the sensitive volume on the model outcomes, we tested three different kinds of shape variations. Keeping a constant length, we let the radius vary, taking up the standard values associated with the other cell lines (4.9, 5.9, 7.0 μm). Similarly, for a fixed radius, we tested a significant increase of the sensitive volume thickness by setting it to the value of the radius itself; in this way, we took into account one of the highest reasonable $L_{V_{s}}$ values in the case of flattened cells. Finally, we performed a compression or a distension along the track, keeping the same $V_{s}$; practically, we chose a value for the radius and derived the length accordingly, under the constant volume constraint (see Table 2).

### 3.4. Effective Lethal Function

**Figure 1:**
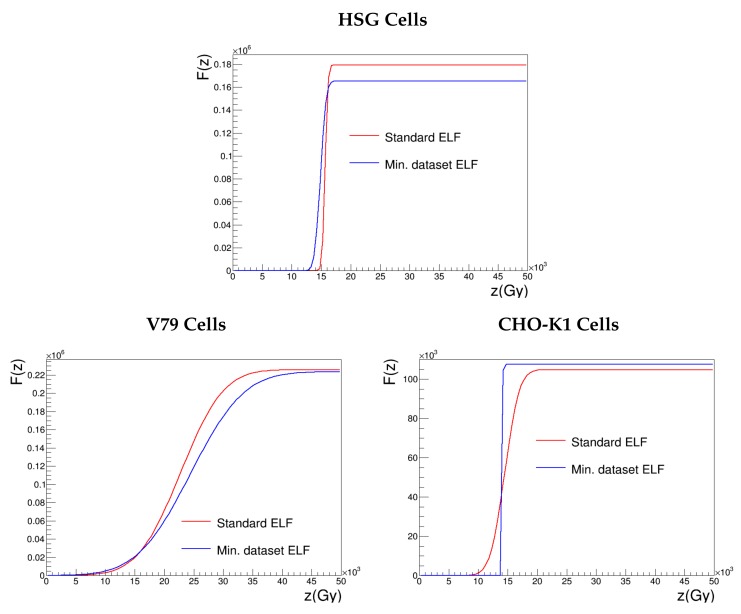
Effective lethal functions optimized considering the standard dataset or the minimal dataset; the latter consists of α(LET) values corresponding to irradiations with photons, one carbon ion with LET between 55 and 75 keV μm^−1^ and one carbon ion with LET between 150 and 200 keV μm^−1^.

We decided to study the sensitivity of the effective lethal function to a given input dataset instead of testing separate variations of the parameters $z_{0}$, σ and *h*, which cannot be fitted independently. In this way, we obtained a modified ELF, still physically relevant and not built up ad hoc. To perform a beneficial analysis from a clinical point of view, we tested therefore a minimal dataset composed of photons and two carbon ions of intermediate and high-LET values. Since the choice of the three experimental points was based on the data available in the literature, we could not work with exactly the same dataset for all the cell lines, but depending on the reported α values, we defined two LET ranges: intermediate LET values ranging from 55–75 keV μm^−1^ and high-LET values ranging from 150–200 keV μm^−1^. Figure 1 allows one to appreciate the closeness of the shapes of the effective lethal functions obtained optimizing the agreement with the standard and the minimal datasets (respectively, 11–14 and 3 experimental points). Table 3 and Table 4, on the other hand, elucidate the list of considered input ions and output parameters in the two cases.

### 3.5. Time of ^•^OH Radical Diffusion, $T_{\mathrm{RCE}}$

**Figure 2:**
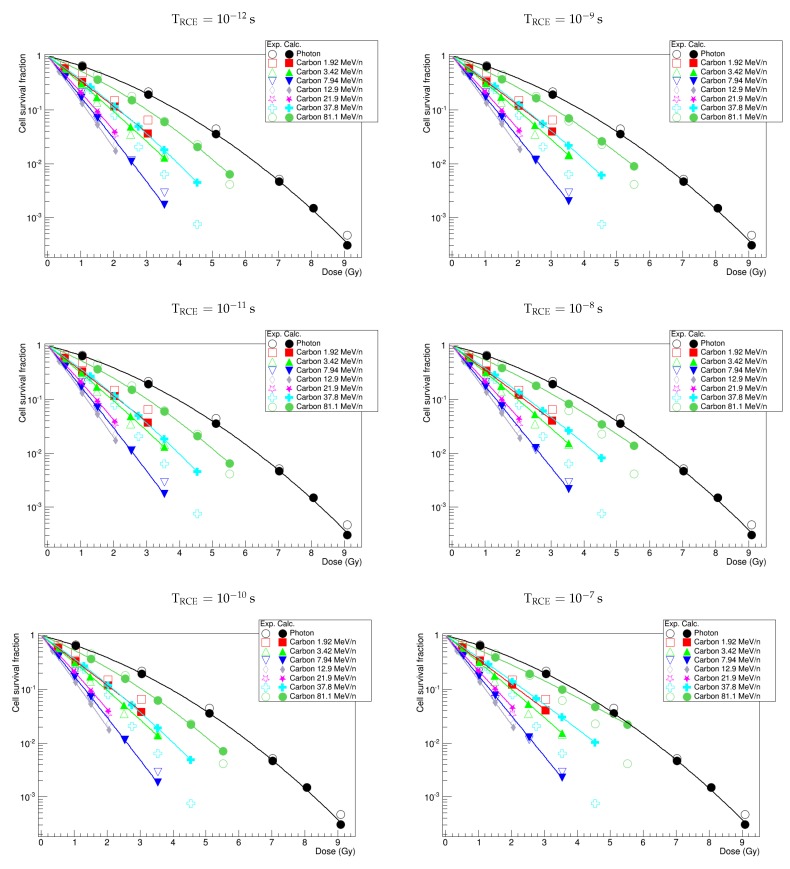
Calculated (Calc.) survival curves of HSG cells irradiated with photons and carbon ions compared to experimental data (Exp.) issued from [25]. In each graph, the probability of cell survival was calculated by assigning a different value to $T_{\mathrm{RCE}}$.

Reactive chemical species are produced $10^{-12}$ s after the interaction between incident ions and biological matter and rapidly either are scavenged or induce some indirect DNA damages. At the preliminary stage, in order to assess how NanOx predictions are influenced by the evolution of ^•^OH radical yield with time, we estimated cell survival curves for different instants, up to $10^{-7}$ s. Figure 2, representing HSG cells irradiated by photons and carbon ions of various energies, shows qualitatively that the amplitude of the shoulder increases with $T_{\mathrm{RCE}}$ and that this phenomenon is particularly visible for low-LET ions at high dose values. To quantify more precisely the role of $T_{\mathrm{RCE}}$ on cell survival modeling, we compare in the following the results obtained with the standard value of $T_{\mathrm{RCE}}=10^{-11}$ s and with $T_{\mathrm{RCE}}=10^{-8}$ s; we consider, indeed, that the latter value represents a significantly different scenario regarding the spatial distribution of ^•^OH radicals.

### 3.6. Quadratic Coefficient for the Reference Radiation, $\beta_{r}$

To test the impact of $\beta_{r}$ on NanOx predictions, we considered different variations for each cell line according to the experimental dispersion found in the literature. We excluded the extreme and rare values and took into account the measurements that reproduced the extension of the cloud of experimental data, being very distant from the average. Table 5 illustrates the choice of the standard and of the varied $\beta_{r}$ for the three cell lines under study.

## 4. Results and Discussion

We stress again that in order to assess the effect induced by the variation of one single parameter at a time, we considered two different outcomes calculated by NanOx as a function of LET: on the one hand, the linear coefficient α, which is widely adopted both experimentally and theoretically to quantify the biological effect of ions; on the other, $D_{x\%}$, the dose deposited to achieve *x*% of cell survival, which is more relevant to clinicians. We set x=1 for the parameters essentially affecting the shoulder of cell survival curves, and x=10 for all the others.

### 4.1. Sensitive Volume, $V_{s}$

The geometry of the sensitive volume is determined from experimental data, which possibly may not exist for a specific cell line or vary significantly from one publication to another. It is therefore useful to survey how NanOx predictions depend on the sensitive volume radius, length and shape.

**Figure 3:**
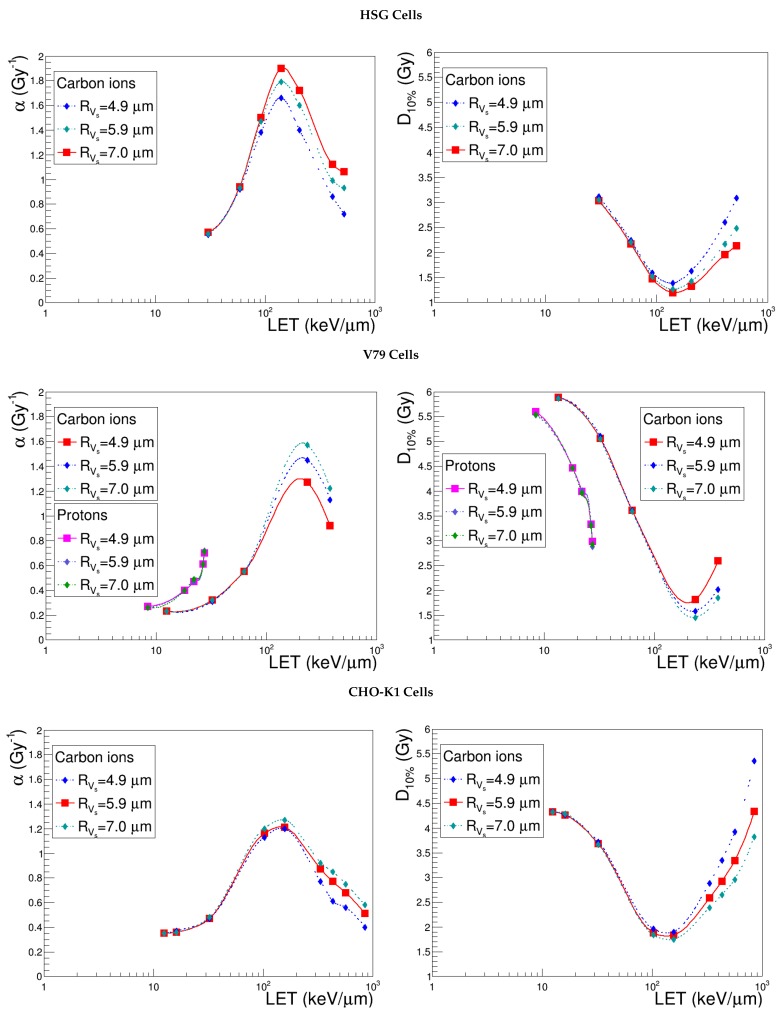
α and $D_{10\%}$ predicted by NanOx for the standard configuration (red curve) and for the varied sensitive volume radii (blue and light blue curves) keeping a constant thickness, as detailed in Table 2. The full symbols correspond to the LET values for which the estimates were performed, while the lines are drawn just to guide the eye and do not represent a fit.

Figure 3 shows the evolution of α and $D_{10\%}$ with LET for three different $V_{s}$ radii, provided the same standard thickness. The simulations describe the cellular responses to carbon ions and, in the case of V79 cells, also protons. We observe, first of all, that the influence of the $V_{s}$ radius is almost independent of the specific cell line. Indeed, when considering a common LET range (30 keV μm^−1^ < LET < 435 keV μm^−1^), the largest variations obtained on α and $D_{10\%}$ by shifting the $V_{s}$ radius from 4.9–7.0 μm are of the same order of magnitude for HSG (26.9% and 28.5%), V79 (36.5% and 33.1%) and CHO-K1 cells (32.7% and 23.4%). Moreover, we notice that the increase of the $V_{s}$ radius increases the cell killing efficiency for high-LET ions, while it does not play a role in the case of low-LET ions, whose radiation impacts are more numerous and more homogeneously distributed. To analyze the behavior of the linear coefficient, let us first recall that:

(12) $$\alpha =\lim_{D\to 0}\left(-\frac{\ln[S(D)]}{D}\right)$$

Considering that D=F·LET·c, where *F* represents the beam fluence and *c* a conversion factor equal to 0.1602 Gy keV^−1^ μm^3^, we may actually take the low fluence limit into account. Since the probability that an incident particle hits the sensitive volume (*P*) corresponds to the product of the beam fluence and the sensitive volume area ($F\;\cdot \;V_{s}/L$), the cell surviving fraction may be expressed as:

(13) $$\lim_{F\to 0}S=1-P+P\;\cdot \;S_{L,1}=\left(1-F\;\cdot \;\frac{V_{s}}{L}\right)+F\;\cdot \;\frac{V_{s}}{L}\;\cdot \;S_{L,1}$$

where $S_{L,1}$ represents the survival with respect to one impact generated only by local lethal events. Equation (13) holds thanks to the null value of $\alpha_{G}$ in the current implementation of the model and in the approximation that $\beta_{G}{\tilde{Z}}^{2}$ is negligible. Let us develop $S_{L,1}$ in a Taylor series, referring to the general definition for a configuration of impacts $c_{K}$ (Equation (9)); for very low fluence values, all the terms of second and higher order may be neglected. At this stage, we may examine the two extreme LET ranges in order to estimate the dependence of α on the sensitive volume radius. In the low-LET region:

(14) $$\lim_{\begin{array}{c} F\to 0\\ LET\to 0 \end{array}}S=\left(1-F\;\cdot \;\frac{V_{s}}{L}\right)+F\;\cdot \;\frac{V_{s}}{L}\;\cdot \;\left(1-\frac{\epsilon }{V_{s}}\right)=1-\frac{F}{L}\epsilon$$

where $\epsilon =({}^{t_{K}}\alpha_{c}V_{c}{}^{t_{K}}Z_{c}+{}^{c_{K}}\alpha_{p}V_{p}{}^{c_{K}}Z_{p})$ is much smaller than one. The fact that Equation (14) is independent of the sensitive volume explains why the α curves obtained with different $V_{s}$ radii superimpose in the low-LET region. For very high-LET values, on the other hand, the cell survival fraction to one impact would be approximately null, so one may derive:

(15) $$\lim_{\begin{array}{c} F\to 0\\ LET\to \infty \end{array}}S=1-F\;\cdot \;\frac{V_{s}}{L}$$

Hence, the variation of the sensitive volume radius in the overkill region is directly propagated to α. The explanation of the effect on $D_{10\%}$ is not straightforward, since it also involves parameter β, which is estimated via the cell survival probability with respect to global events (Equation (10)). The non-linear term appearing in the exponential complicates the calculations, and the low fluence approximation cannot be exploited in this case. We may, however, make some general considerations to infer the variations of the non-linear component of cell survival with respect to the $V_{s}$ radius in the extreme LET regions. Photon irradiations may represent the very low-LET values; in this case, the dependence of $S_{G}$ on the sensitive volume is expressed uniquely via the distribution of the restricted specific energy. However, it has been shown [31] that whenever $V_{s}$ has dimensions comparable to those of cell nuclei or greater, the restricted specific energy obeys a Gaussian-like distribution peaked at a value that is independent of the target volume. We can hypothesize that the same conclusion holds for low-LET ions, whose surviving fractions with respect to global events approach the ones of photons. It is not surprising, hence, that the $D_{10\%}$ curves obtained by varying the $V_{s}$ radius from 4.9 to 7 μm are superimposed in this case. On the other hand, in the limit of very high-LET values, the distribution of restricted specific energy is much more heterogeneous, and in this context, the influence of the sensitive volume radius becomes manifest.

**Figure 4:**
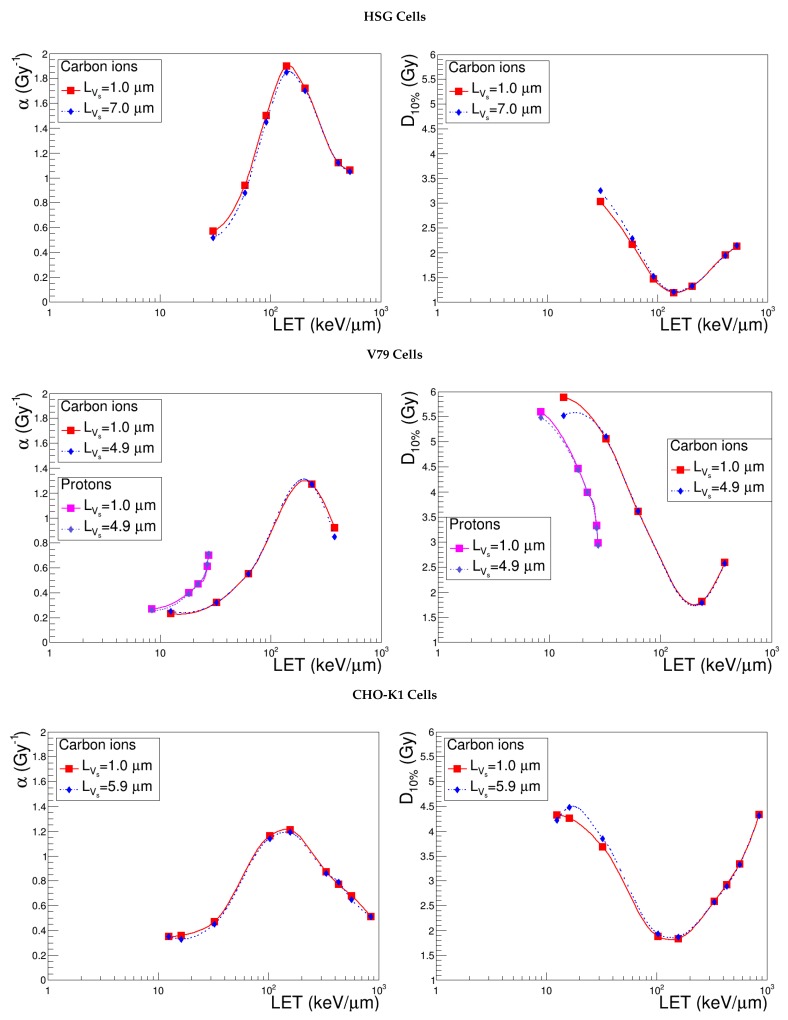
α and $D_{10\%}$ predicted by NanOx for the standard configuration (red curve) and for the varied sensitive volume length (blue curve) keeping the standard radius, as detailed in Table 2. The full symbols correspond to the LET values for which the estimates were performed, while the lines are drawn just to guide the eye and do not represent a fit.

Figure 4 discloses the evolution of α and $D_{10\%}$ curves calculated with different values of the $V_{s}$ length and shows, at first sight, that the parameter under study has a negligible impact. In this case, the sensitivity study cannot be carried out simply by considering the maximum relative difference obtained for α and $D_{10\%}$ in a common LET range; indeed, different input variations on $L_{V_{S}}$ were associated with the three cell lines (600% for HSG, 390% for V79 and 490% for CHO-K1). We opted therefore to plot Δα/α (resp. $\Delta D_{10\%}/D_{10\%}$) as a function of $\Delta L_{V_{s}}/L_{V_{s}}$ and inferred that the points corresponding to the three cell lines were linearly correlated. We deduced, hence, that NanOx’s sensitivity to the variation of the sensitive volume length is independent of the cell and extremely low: the maximum relative difference obtained for α ($D_{10\%}$) in the common LET range is 10.0% (7.3%) when $L_{V_{s}}$ is made to vary from 1–7 μm. As a conclusion, we can state that the length of the cylinder representing the sensitive volume is a “second level” parameter, which may be fixed to the standard value for all the cell lines.

**Figure 5:**
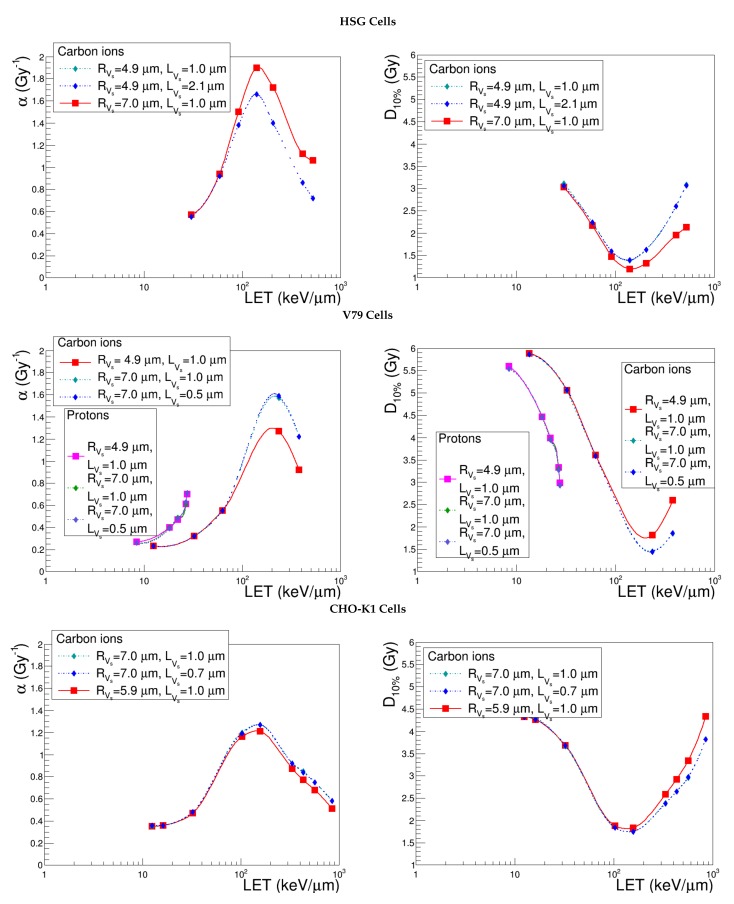
α and $D_{10\%}$ predicted by NanOx for the standard configuration (red curve), a different sensitive volume shape (blue curve) and radius (light blue curve), as detailed in Table 2. The full symbols correspond to the LET values for which the estimates were performed, while the lines are drawn just to guide the eye and do not represent a fit.

In order to survey the effect induced by a deformation, the radius and the length were also made to vary under the constraint of the constant sensitive volume. Figure 5 shows that NanOx predictions are affected by the compression or distension of microtargets in the direction of the track. However, it is worth noting that this is almost uniquely due to the reduction or increase of the $V_{s}$ radius: the α and $D_{10\%}$ curves superimpose with the ones obtained with the same radius, but keeping the standard thickness of 1 μm, which were drawn for completeness, confirming the previous conclusion on $L_{Vs}$. Since these variations were performed preserving the standard configuration of the effective lethal function, the total number and the density of nanometric targets in the sensitive volume is constant. Thus, for a given ion energy, the probability of inactivating a nanotarget is constant for a fixed $V_{s}$ radius, whatever the $V_{s}$ length. Note that switching, for instance, from a cylindrical sensitive volume to a spherical one would change the predictions; a sphere can be interpreted as a stacking of thin cylinders, the radii of which depend on the position along the ions beam.

Finally, we stress that whenever the effective lethal function is optimized according to the sensitive volume geometry, the impact of such a geometry is strongly reduced. This procedure (not described in the paper) was performed for HSG cells and showed that even the prediction accuracy of the $V_{s}$ radius is not critical because of the compensation issued from the ELF re-tuning. This may have important implications in view of a clinical application of the model.

### 4.2. Effective Lethal Function

The determination of the effective lethal function depends on the set of experimental data used to fit its parameters. It is important to know, in particular to comply with the demands of the clinical environment, if reducing this set of experimental data would severely degrade the quality of the predictions.

**Figure 6:**
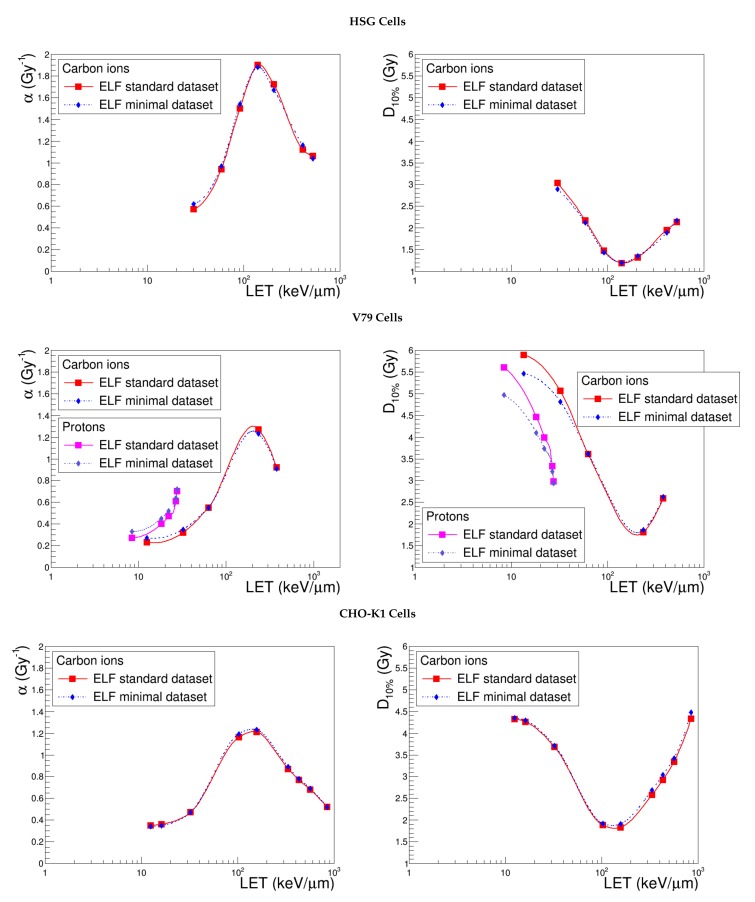
α and $D_{10\%}$ predicted by NanOx for the standard configuration (red curve) and optimizing the effective lethal function with only three data points (blue curve). The full symbols correspond to the LET values for which the estimates were performed, while the lines are drawn just to guide the eye and do not represent a fit.

Figure 6 illustrates α and $D_{10\%}$ obtained with NanOx by optimizing the effective lethal functions of the three cell lines according to the standard dataset and to a subset of three experimental points. We observe that, globally, the determination of an alternative set of ELF parameters in a “clinician-oriented scenario” has almost no impact on NanOx predictions for intermediate and high-LET ions. This is, of course, intrinsically related to the method used to constitute the alternative dataset, which aims at achieving an accurate description of the Bragg peak optimizing the treatment in the tumoral region. With an input dataset constituted by the experimental α value of photons and two carbon ions with LET in the ranges (55–75) keV μm^−1^ and (150–200) keV μm^−1^, the modeling of the biological effects induced by low-LET ions, on the contrary, is less satisfying. Some discrepancies are visible, in particular for V79 cells, for which we proved that the proportion of local lethal events arising from the core region is greater than for the other cell lines. Let us recall that only the description of this kind of event relies on the effective lethal function; as detailed in Section 2.3, the coefficient $t_{k}\alpha_{c}$ is determined thanks to the ELF, while ${}^{c_{k}}\alpha_{p}$ is approximated by $\alpha_{r}$. Depending on the purpose, therefore, one should prefer a certain kind of input measurements (e.g., low-LET ions to estimate the radiation-induced effects in the healthy tissues). In any case, these results look promising since they testify to the robustness of NanOx and underline the feasibility of a clinical application in a realistic context of a scarcity of experimental data.

Besides, this study motivates some speculations regarding the role of the ELF parameters, even if it should be carried out with more cell lines to draw firm conclusions. Table 4 shows that relative variations as big as 50% and 98% for σ do not affect the output in a significant way; the width of the increase of the error function seems therefore to be a “second level” parameter. Changing the input dataset, instead, induces relative variations lower than 5% and 8% for $z_{0}$ and *h*; this suggests a larger relevance of these parameters in the modeling of cell survival with respect to local lethal events.

### 4.3. Time of ^•^OH Radical Diffusion, $T_{\mathrm{RCE}}$

The calculation of cell survival with respect to global events relies on the estimate of the chemical specific energy, which aims at representing the oxidative stress induced by the ionizing particles. In the current version of Nanox, this stress is induced by the production of ^•^OH radicals in the sensitive volume. Due to the process of diffusion and to the chemical reactions that take place, however, the concentration of ^•^OH radicals depends on the time interval separating the impact of the incident particles and the moment at which this concentration is considered.

**Figure 7:**
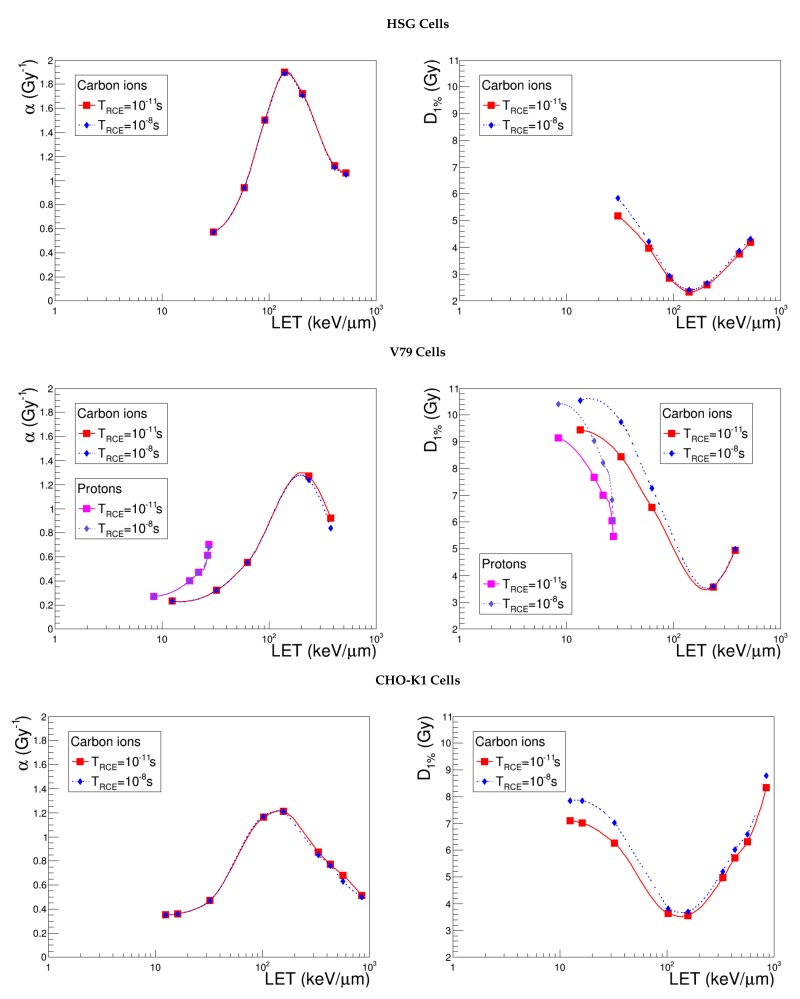
α and $D_{1\%}$ predicted by NanOx for the standard configuration (red curve) and for $T_{\mathrm{RCE}}=10^{-8}s$ (blue curve). The full symbols correspond to the LET values for which the estimates were performed, while the lines are drawn just to guide the eye and do not represent a fit.

**Figure 8:**
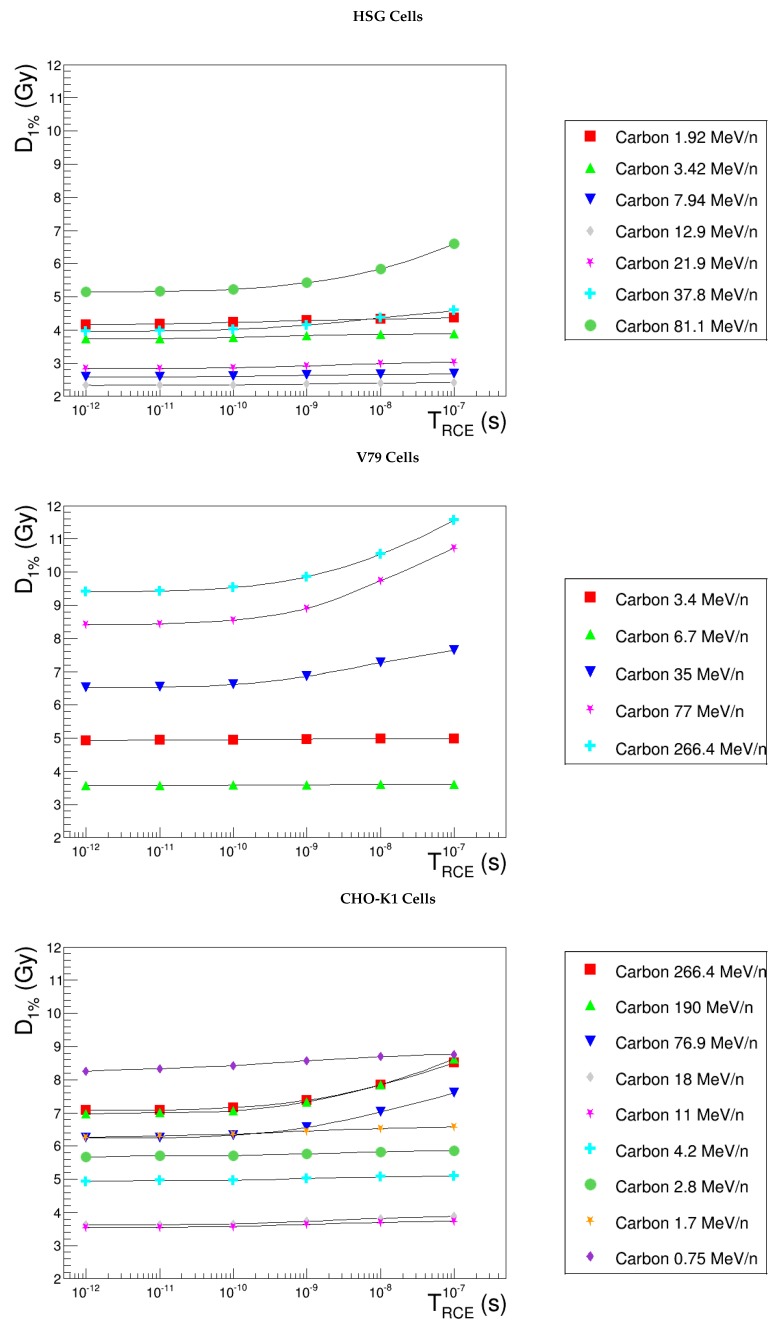
$D_{1\%}$ predicted by NanOx as a function of the time of ^•^OH radical diffusion, $T_{\mathrm{RCE}}$. The full symbols correspond to the LET values for which the estimates were performed, while the lines are drawn just to guide the eye and do not represent a fit.

In order to estimate the impact of $T_{\mathrm{RCE}}$ on NanOx predictions, the evolution of α and $D_{1\%}$ with LET was evaluated (see Figure 7) for two distant values of time: $10^{-11}$ s and $10^{-8}$ s. We observe that the linear component of the cell survival curve is not affected by $T_{\mathrm{RCE}}$. This implies that the study of $D_{1\%}$ allows one to focus essentially on the impact of time on the non-linear part of the cell survival. When switching $T_{\mathrm{RCE}}$ from $10^{-11}$ s to $10^{-8}$ s, indeed, the increase of β becomes manifest in the low-LET range, and as a consequence, $D_{1\%}$ decreases. This effect is not exhibited by high-LET ions, for which the shoulder is less important. Considering a common LET range for the three cell lines, the extreme $D_{1\%}$ relative differences are found to be 13.1% for HSG cells, 12.4% for CHO-K1 and 15.4% for V79 cells. These results highlight two main conclusions: the impact of $T_{\mathrm{RCE}}$ on NanOx predictions is limited and almost independent of the cell line. Such arguments motivate the idea of fixing time to a convenient value, reducing the number of free parameters. We performed, hence, a further analysis to investigate the evolution of $D_{1\%}$ as a function of time. Figure 8 shows this observable from $T_{\mathrm{RCE}}=10^{-12}$ s–$T_{\mathrm{RCE}}=10^{-7}$ s: the curves associated with various carbon ions are almost constant with respect to time, except for a slight increase, which is observable for low-LET ions and $T_{\mathrm{RCE}}>10^{-10}$ s. As a consequence, from now on, we consider that the time $T_{\mathrm{RCE}}$ represents a “second level” parameter, which may be fixed to the standard value of $10^{-11}$ s for all the cell lines. This time `tick’ corresponds to the primary production of radicals just after the very fast reactions involving the chemical species that are almost in contact. The recombination process is more important for the high-LET ions, as the concentration of ionizations and molecular excitations is higher in that case.

### 4.4. Quadratic Coefficient for the Reference Radiation, $\beta_{r}$

**Figure 9:**
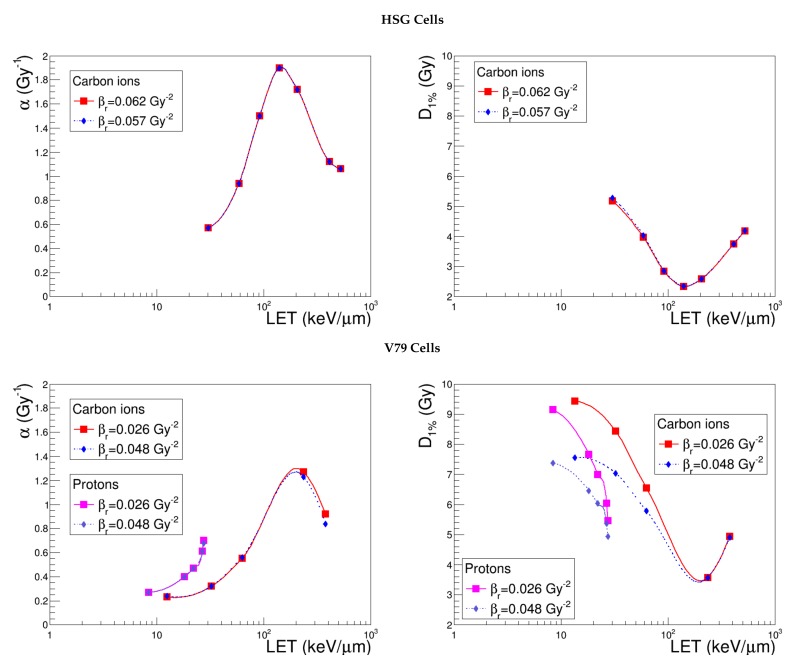

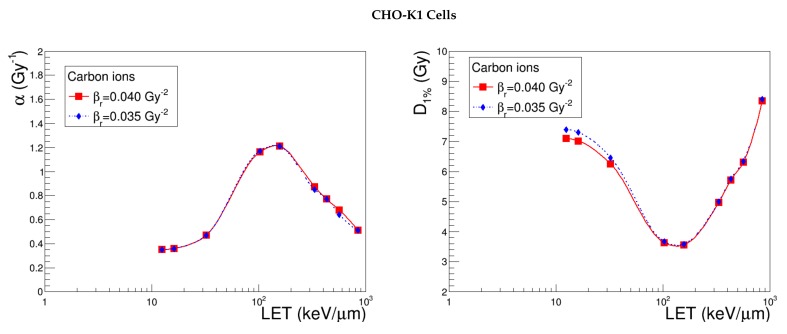
α and $D_{1\%}$ predicted by NanOx for the standard configuration (red curve) and for different $\beta_{r}$ values (blue curve), as detailed in Table 5. The full symbols correspond to the LET values for which the estimates were performed, while the lines are drawn just to guide the eye and do not represent a fit.

It is known that the measurement of the quadratic coefficient β is characterized by a high variability. Since this experimental value for the reference radiation, $\beta_{r}$, affects the cell survival fractions calculated by NanOx for every radiation type, it is of great interest to examine the effect of the uncertainty related to it. Figure 9 shows α and $D_{1\%}$ calculated as a function of LET with the standard $\beta_{r}$, and with a value chosen in a range that reproduces the cloud of experimental data corresponding to each cell line. By comparing the curves obtained for the two observables, it is possible to deduce that this parameter almost exclusively affects the non-linear component of cell survival and is especially important for low-LET ion irradiations. However, to evaluate NanOx’s sensitivity to its variation accurately, one should take into account the very different $\beta_{r}$ shifts which were associated with the three cell lines in order to reproduce the realistic dispersion of the experimental measurements (8.5% for HSG, 85% for V79 and 12.5% for CHO-K1). We plotted the output $\Delta D_{1\%}/D_{1\%}$ as a function of the input $\Delta \beta_{r}/\beta_{r}$ and realized that the values corresponding to the three cell lines display the same linear relation. This result underlines that, while the nominal value chosen to characterize $\beta_{r}$ is by definition cell line specific, the impact of the variation of such a parameter is almost independent of the cell line. Furthermore, $D_{1\%}$ shows a weak sensitivity to the dispersion of the experimental $\beta_{r}$ measurements.

### 4.5. Towards a Clinical Application

The possibility to fix the value of $L_{V_{s}}$ and $\beta_{r}$ for any cell line without degrading the result, and the study on the minimal dataset required to fit the effective lethal function, proved that NanOx requires only a few experimental data to predict cell survival probability to ion irradiations. This opens promising perspectives for its potential clinical application, such as the optimization of a personalized therapy. In the future, we may hypothesize determining the average nuclear radius of tumoral cells on a biopsy sample and to irradiate the latter with photons and two carbon ion beams of intermediate and high-LET; this would allow one to measure $\alpha_{r}$, $\beta_{r}$ and the two α coefficients for the carbon ions. With these five input items, NanOx could be employed to optimize the individual patient prescription, when implemented in a treatment planning system. This promising scenario incites us to perform further studies in order to test the model predictions in conditions that are closer to the clinical context. For this reason, we plan to extend our research field to spread-out Bragg peak irradiations and to other cell lines matching the therapeutic indications for hadron therapy treatment. On the other hand, we intend to consider non-tumoral cells to evaluate the normal tissue damage and early responding tissues characterized by a high α/β ratio, since for the considered cell lines, the latter varies from 3.2–5.5 Gy.

## 5. Conclusions

This work provides a detailed discussion of the sensitivity of NanOx predictions to most of its parameters. Each of them is made to vary independently of the others, and the effect is assessed via the analysis of three different outcomes for HSG, V79 and CHO-K1 cells in response to proton and carbon ion irradiations. This study demonstrates that in the current version of NanOx, the prediction of the biological effect of ions for a wide LET range may be based on only five parameters characterizing a given cell line. The cellular region where the biological damage is supposed to be achieved both at local and global scales is entirely described in terms of the sensitive volume radius, $R_{V_{s}}$. The modeling of local lethal events taking place at a nanometric scale relies on the three parameters defining the ELF: while σ, the width of the increase, may be significantly changed without altering the considered endpoints, $z_{0}$ and *h*, respectively representing the function’s threshold and amplitude of the response, seem more critical since they considerably affect NanOx output. Finally, it is possible to reproduce the shoulder appearing in the experimental cell survival curves for a variety of ions using a single parameter, the quadratic coefficient associated with photon irradiations, $\beta_{r}$. The sensitivity analysis highlights that the time of ^•^OH radical diffusion and the length of the cylindrical sensitive volume may be fixed for any of the considered cell lines to some standard values.

This work also sheds light on the input data required to calculate cell survival probability with respect to ion irradiations. In particular, the experimental evolution of the linear parameter with LET may be retrieved in a good approximation thanks to an ELF optimized with only three α values measured for photons and carbon ions of energy between (8–12) MeV u^−1^ and (25–40) MeV u^−1^. This result opens interesting perspectives from a clinical point of view, since NanOx predictions rely only on a few experimental measurements: the average cellular nuclear radius, the linear quadratic coefficients describing photon irradiations and the α values associated with two carbon ions with intermediate and high-LET values.

## Figures and Tables

**Table 1:** Standard values of the parameters used to model the three cell lines with NanOx. $R_{V_{s}}$ (resp. $L_{V_{s}}$) represents the sensitive volume radius (length) and, similarly, $R_{V_{t}}$ (resp. $L_{V_{t}}$) the local targets radius (length).

Class	Parameters	Variations	Standard Values	Standard Values	Standard Values
		Considered	for HSG Cells	for V79 Cells	for CHO-K1 Cells
Local/Global	$R_{V_{s}}$ (μm)	✓	7.0	4.9	5.9
	$L_{V_{s}}$ (μm)	✓	1.0	1.0	1.0
Local	$z_{0}$ (Gy)	✓	15,654	22,789	14,507
	σ (Gy)	✓	549	8117	2781
	*h*	✓	179,439	225,841	104,810
	$R_{V_{t}}$ (nm)	✗	10		
	$L_{V_{t}}$ (nm)	✗			
Global	$\beta_{G}$ (Gy^−2^)	✓	0.0961	0.0405	0.0625
	$\alpha_{G}$ (Gy^−1^)	✗	0		
	$T_{\mathrm{RCE}}$ (s)	✓	$10^{-11}$	$10^{-11}$	$10^{-11}$

**Table 2:** Input variations of sensitive volume radius and thickness for the three cell lines.

Cell Line	Standard $V_{s}$		$V_{s}$ Radius Var. 1		$V_{s}$ Radius Var. 2		$V_{s}$ Length Var.		$V_{s}$ Shape Var.	
	R (μm)	L (μm)	R (μm)	L (μm)	R (μm)	L (μm)	R (μm)	L (μm)	R (μm)	L (μm)
**HSG**	7.0	1.0	5.9	1.0	4.9	1.0	7.0	7.0	4.9	2.1
**V79**	4.9	1.0	5.9	1.0	7.0	1.0	4.9	4.9	7.0	0.5
**CHO-K1**	5.9	1.0	4.9	1.0	7.0	1.0	5.9	5.9	7.0	0.7

**Table 3:** Standard and minimal experimental datasets used as input to optimize the effective lethal function of each cell line.

Cell Line	Standard Dataset		Minimal Dataset	
	Particle Type	Energies (MeV u^−1^)	Particle Type	Energies (MeV u^−1^)
**HSG**	Photons		Photons	
	Helium ions	2.9, 5.6, 8.4	Carbon ions	8.5, 39.1
	Carbon ions	2.3, 8.5, 21.4, 39.1, 82.6		
	Neon ions	17.2, 43.6, 84.4, 96.4		
**V79**	Photons		Photons	
	Protons	2.6, 7.7	Carbon ions	12, 28.4
	Helium ions	2.9, 9.2		
	Carbon ions	12, 28.4, 67.6, 190		
	Neon ions	23, 47.8, 105		
	Argon ions	17.3, 46.5, 170		
**CHO-K1**	Photons		Photons	
	Carbon ions	2.4, 4.8, 11, 35	Carbon ions	11, 35
	Neon ions	1.7, 11, 28.7, 58		
	Oxygen ions	11, 141.5, 360		

**Table 4:** Output parameters obtained by optimizing the lethal function of each cell line with the standard and minimal datasets; the relative differences are also reported.

Cell Line	Standard Dataset,	Minimal Dataset	Relative
	Output Parameters	Output Parameters	Variation (%)
**HSG**	$z_{0}$ = 15,654	$z_{0}$ = 14,858	5
	σ = 549	σ = 1063	50
	*h* = 179,439	*h* = 165,283	8
**V79**	$z_{0}$ = 22,789	$z_{0}$ = 24,481	7
	σ = 8117	σ = 10,135	25
	*h* = 22,5841	*h* = 22,3597	1
**CHO-K1**	$z_{0}$ = 14,507	$z_{0}$ = 14,191	2
	σ = 2781	σ = 44	98
	*h* = 104,810	*h* = 107,548	3

**Table 5:** Standard and varied $\beta_{r}$ values chosen for each cell line, with the corresponding relative differences. The numbers in square brackets are the reference publications.

Cell Line	Standard $\beta_{r}$ (Gy^−2^),	Varied $\beta_{r}$ (Gy^−2^),	Relative
	(Source)	(Source)	Difference (%)
**HSG**	0.0615	0.0565	8.5
	[25]	[26]	
**V79**	0.0259	0.0480	85.3
	[27]	[28]	
**CHO-K1**	0.0200 × 2	0.0350	12.5
	[29]	[30]	

## References

[1] Wedenberg M., Lind B.K., Hardemark B. (2013). A model for the relative biological effectiveness of protons: The tissue specific parameter *α*/*β* of photons is a predictor for the sensitivity to LET changes. Acta Oncol..

[2] McNamara A.L., Schuemann J., Paganetti H. (2015). A phenomenological relative biological effectiveness (RBE) model for proton therapy based on all published in vitro cell survival data. Phys. Med. Biol..

[3] Carante M.P., Ballarini F. (2016). Calculating Variations in Biological Effectiveness for a 62 MeV Proton Beam. Front. Oncol..

[4] Krämer M., Scholz M. (2000). Treatment planning for heavy-ion radiotherapy: Calculation and optimization of biologically effective dose. Phys. Med. Biol..

[5] Krämer M., Scifoni E., Wälzlein C., Durante M. (2012). Ion beams in radiotherapy—From tracks to treatment planning. J. Phys..

[6] Endo M., Koyama-Ito H., Minohara S.I., Miyahara N., Tomura H., Kanai T., Kawachi K., Tsujii H., Morita K. (1996). HIPLAN—A Heavy Ion Treatment Planning System at HIMAC. J. JASTRO.

[7] Inaniwa T., Furukawa T., Kase Y., Matsufuji N., Toshito T., Matsumoto Y., Furusawa Y., Noda K. (2010). Treatment planning for a scanned carbon beam with a modified microdosimetric kinetic model. Phys. Med. Biol..

[8] Beuve M. (2009). Formalization and theoretical analysis of the Local Effect Model. J. Radiat. Res..

[9] Russo G., Attili A., Bourhaleb F. (2011). Analysis of the reliability of the local effect model for the use in carbon ion treatment planning systems. Radiat. Prot. Dosim..

[10] Cunha M., Monini C., Testa E., Beuve M. (2017). NanOx, a new model to predict cell survival in the context of particle therapy. Phys. Med. Biol..

[11] Monini C., Testa E., Beuve M. (2017). NanOx predictions of cell survival probabilities for three cell lines. Acta Phys. Pol. Ser. B.

[12] Monini C., Testa E., Beuve M. (2018). Positioning of NanOx among the biophysical models for hadron therapy. Radiat. Prot. Dosim..

[13] Beuve M., Colliaux A., Dabli D., Dauvergne D., Gervais B., Montarou G., Testa E. (2009). Statistical effects of dose deposition in track-structure modelling of radiobiology efficiency. Nucl. Instrum. Methods Phys. Res. Sect. B.

[14] Gervais B., Beuve M., Olivera G., Galassi M., Rivarola R. (2005). Production of HO_2_ and O_2_ by multiple ionization in water radiolysis by swift carbon ions. Chem. Phys. Lett..

[15] Ravanat J.L., Douki T., Cadet J. (2001). Direct and indirect effects of UV radiation on DNA and its components. J. Photochem. Photobiol. B.

[16] Von Sonntag C. (2006). Free-Radical-Induced DNA Damage and Its Repair.

[17] Gervais B., Beuve M., Olivera G., Galassid M.E. (2006). Numerical simulation of multiple ionization and high LET effects in liquid water radiolysis. Radiat. Phys. Chem..

[18] Colliaux A., Gervais B., Rodriguez-Lafrasse C., Beuve M. (2015). Simulation of ion-induced water radiolysis in different conditions of oxygenation. Nucl. Instrum. Methods Phys. Res. Sect. B.

[19] Alpen E.L. (1997). Radiation Biophysics.

[20] James F. (1994). MINUIT Function Minimization and Error Analysis: Reference Manual Version 94.1.

[21] Elsässer T., Scholz M. (2007). Cluster effects within the Local Effect Model. J. Radiat. Res..

[22] Schipler A., Iliakis G. (2013). DNA double-strand—Break complexity levels and their possible contributions to the probability for error-prone processing and repair pathway choice. Nucleic Acids Res..

[23] Nikjoo H., O’Neill P., Goodhead D.T., Terrissol M. (1997). Computational modelling of low-energy electron-induced DNA damage by early physical and chemical events. Int. J. Radiat. Biol..

[24] Kreipl M.S., Friedland W., Paretzke H.G. (2009). Interaction of ion tracks in spatial and temporal proximity. Radiat. Environ. Biophys..

[25] Furusawa Y., Fukutsu K., Aoki M., Itsukaichi H., Eguchi-Kasai K., Ohara H., Yatagai F., Kanai T., Ando K. (2000). Inactivation of aerobic and hypoxic cells from three different cell lines by accelerated (3)He-, (12)C- and (20)Ne-ion beams. J. Radiat. Res..

[26] Aoki-Nakano M., Furusawa Y., Uzawa A., Matsumoto Y., Hirayama R., Tsuruoka C., Ogino T., Nishio T., Kagawa K., Murakami M. (2014). Relative biological effectiveness of therapeutic proton beams for HSG cells at Japanese proton therapy facilities. J. Radiat. Res..

[27] Cox R., Thacker J., Goodhead D.T. (1977). Inactivation and Mutation of Cultured Mammalian Cells by Aluminium Characteristic Ultrasoft X-rays: II. Dose-responses of Chinese Hamster and Human Diploid Cells to Aluminium X-rays and Radiations of Different LET. Int. J. Radiat. Biol..

[28] Folkard M., Prise K., Voijnovic B., Newman H.C., Roper M.J., Michael B.D. (1996). Inactivation of V79 cells by low-energy protons, deuterons and helium-3 ions. Int. J. Radiat. Biol..

[29] Weyrather W., Ritter S., Scholz M., Kraft G. (1999). RBE for carbon track-segment irradiation in cell lines of differing repair capacity. Int. J. Radiat. Biol..

[30] Hill M.A., Herdman M.T., Stevens D.L., Jones N.J., Thacker J., Goodhead D.T. (2004). Relative Sensitivities of Repair-Deficient Mammalian Cells for Clonogenic Survival after *α*-Particle Irradiation. J. Radiat. Res..

[31] Cunha M., Testa E., Beuve M., Balosso J., Chaikh A. (2017). Considerations on the miniaturization of detectors for in vivo dosimetry in radiotherapy: A Monte Carlo study. Nucl. Instrum. Methods Phys. Res. B.

